# The putative pleiotropic functions of meprin β in gastric cancer

**DOI:** 10.1007/s10120-023-01385-2

**Published:** 2023-03-28

**Authors:** Wiebke Siemsen, Christine Halske, Hans-Michael Behrens, Sandra Krüger, Christoph Becker-Pauly, Christoph Röcken

**Affiliations:** 1 Department of Pathology, Christian-Albrechts-University, Arnold-Heller-Str. 3, House U33, 24105 Kiel, Germany; 2 Institute of Biochemistry, Christian-Albrechts-University, Kiel, Germany

**Keywords:** Gastric cancer, Meprin β, Microbiome

## Abstract

**Background:**

The gastric microbiome and inflammation play a key role in gastric cancer (GC) by regulating the immune response in a complex manner and by inflammatory events supporting carcinogenesis. Meprin β is a zinc endopeptidase and participates in tissue homeostasis, intestinal barrier function and immunological processes. It influences local inflammatory processes, dysbiosis and the microbiome. Here, we tested the hypothesis that meprin β is expressed in GC and of tumor biological significance.

**Patients and methods:**

Four hundred forty whole mount tissue sections of patients with therapy-naive GC were stained with an anti-meprin β antibody. The histoscore and staining pattern were analyzed for each case. Following dichotomization at the median histoscore into a “low” and “high” group, the expression was correlated with numerous clinicopathological patient characteristics.

**Results:**

Meprin β was found intracellularly and at the cell membrane of GC. Cytoplasmic expression correlated with the phenotype according to Lauren, microsatellite instability and PD-L1 status. Membranous expression correlated with intestinal phenotype, mucin-1-, E-cadherin-, β-catenin status, mucin typus, microsatellite instability, *KRAS* mutation and PD-L1-positivity. Patients with cytoplasmic expression of meprin β showed a better overall and tumor-specific survival.

**Conclusions:**

Meprin β is differentially expressed in GC and has potential tumor biological relevance. It might function as a tumor suppressor or promotor depending on histoanatomical site and context.

## Introduction

Gastric cancer (GC) is the fifth most common cancer in the World with high mortality [1] and poor prognosis [2]. Chronic active gastritis due to *Helicobacter pylori* (HP) infection is the most common risk factor [3–6], and HP has been classified as a type 1 carcinogen by the WHO [3, 7]. Apart from HP, each area of the body has a preferred microbiome and a change in composition can lead to dysbiosis and inflammation [8], which is coupled with genetically determined increased susceptibility to diseases including cancer [3, 8]. A growing body of evidence shows that microbes, the microbiome and inflammation play a key role in tumor progression by regulating the immune response in a complex manner and the inflammatory process supporting carcinogenesis [3, 8]. HP has pro-carcinogenic activity by directly affecting the mucosa of the stomach and promoting DNA damage [3]. By hypermethylating the promoter region, HP inactivates DNA repair, cell adhesion and tumor suppressor genes and consequently promotes cancer development [9]. At this point, it is important to present meprin β. Meprin β is a zinc endopeptidase, which belongs to the group of metalloproteases and participates in tissue homeostasis, intestinal barrier function and immunological processes [10]. It is localised as a dimer on the outer plasma membrane and has cleavage specificity for negatively charged amino acids [11]. Dysregulation is observed in neurodegenerative diseases, inflammation, intestinal diseases and fibrosis [10]. In the small intestine, the influence of meprin β on inflammatory diseases depends on its localisation within the epithelial cell. It has anti-inflammatory activity, when it is localised on the apical side of the epithelial cell and causes the mucus to detach from the mucosa by splitting the mucus. As a result, bacterial overgrowth is prevented [10]. Conversely, mesenchymal localisation causes a proinflammatory stimulus by cleavage of the IL-6 receptor, which as a now soluble IL-6 receptor forms a complex with IL-6 and the glycoprotein gp130 and induces IL-6 trans-signalling [10, 12]. IL-6 trans-signalling promotes proliferation, invasion and metastasis of GC cells [13]. It also induces increased VEGF-C production via the JAK-STAT3 pathway, which results in increased lymphangiogenesis and consequently improves the blood supply to the carcinoma [13]. In addition, meprin β is controlled and activated by multiple enzymes [10]. Primarily, the protease meprin β is in an inactive state on the plasma membrane [14]. There, meprin β is cleaved from the membrane by ADAM10/17 and MT1-MMP [15], a process called ectodomain shedding, and activated by serine proteases [10]. In the detached state, meprin β is involved in procollagen [16] and mucin cleavage [17]. Another possibility is direct activation by matriptase-2 (MT-2) [18]. Once activated, meprin β remains at the plasma membrane where it cleaves the IL-6 receptor and CD99 [10]. There is also a site-dependent function specificity of meprin β here. As a solubilised protease with the task of mucin cleavage, it has an anti-inflammatory effect by preventing bacterial overgrowth. As a membrane-bound protease, it has a proinflammatory effect by releasing the IL-6 receptor via IL-6 trans-signalling and by cleaving CD99 via increased transendothelial cell migration [10, 18]. Meprin β is in an equilibrium of bivalent functions depending on the localisation with a correspondingly different substrate repertoire via the regulation just described, which indicates that meprin β activity must be strictly regulated. Pathogens, such as *Porphyromonas gingivalis* (*P. gingivalis*), a Gram-negative oral anaerobe, can override this balance [19]. *P. gingivalis* secretes the protease Arg-gingipain (RgpB), which converts membrane-bound meprin β into its active form. This activation prevents meprin β shedding, as only one of the two pathways can be taken, and thus impairs the function of meprin β as a mucus-releasing protease [19]. In the absence of mucus detachment, the mucus is no longer loosely attached and rapidly renewed, which would be necessary for efficient intestinal barrier function and prevention of bacterial overgrowth, but instead the mucus is tightly packed and firmly attached to the epithelium [19]. Consequently, the intestinal barrier function is impaired, because the anti-inflammatory effect of meprin β is largely lost, which promotes further bacterial colonisation and dysbiosis of the mucosa [19]. Here, we tested the hypothesis that meprin β is differentially expressed in GC and is of potential tumor biological significance.

## Materials and methods

### Ethics vote

The study was approved by the ethics committee of the University Hospital Schleswig–Holstein Campus Kiel (D453/10, D 525/15).

### Patient collective

The patient collective was retrieved from the archive of the Department of Pathology of the University Hospital Schleswig–Holstein, Campus Kiel. Inclusion criteria for being part of the collective were total or partial gastrectomy due to adenocarcinoma of the stomach or gastro-oesophageal junction without neoadjuvant/perioperative (radio-)chemotherapy. Each resected specimen underwent histological examination by board-certified surgical pathologists, with histological confirmation of the presence of adenocarcinoma. The detection of a tumor type other than adenocarcinoma was an exclusion criterion, as well as neoadjuvant/perioperative (radio-)chemotherapy.

The time of death of the patients was queried at the ‘Epidemiological Cancer Registry Schleswig–Holstein’. Follow-up data were retrieved from hospital records and from the associated general practitioners. All patient data were pseudonymised after inclusion in the study and only evaluated in cumulative form [20].

### Clinicopathological characteristics

Clinicopathological patient characteristics included demographic patient data, anatomical localization, tumor type according to Lauren [21] and TNM classification [22]. Infection with HP was evaluated histologically, using modified Giemsa staining and polymerase chain reaction. HP-specific DNA was detected by a PCR-based assay targeting the 16S rRNA gene of HP*,* as described previously [23]. Epstein–Barr virus-encoded RNA was detected using the EBER probe (Novocastra) and BondMax detection system according to the manufacturer’s instructions (Leica Microsystems GmbH) [24]. Microsatellite instability (MSI) status was assessed by immunohistochemistry using antibodies directed against MLH1, PMS2, MSH2, and MSH6. For each case with reduced or absent nuclear staining, subsequent molecular comparison of the allelic profiles of the mononucleotide repeat markers BAT-25, BAT-26, NR-21, NR-24, and NR-27 in the tumor and corresponding normal tissue was carried out [25]. Furthermore, assessment of *BRAF*- [23], *KRAS*- [23], and *PIK3CA*-genotype [24]; the expression of β-Catenin [26], and E-cadherin [26]; the HER2- [27] and MET status [28], mucin types [23], VISTA [29], and PD-L1 in tumor and immune cells [30], as well as PD-1 in immune cells [30] was done as described in detail previously.

### Histology and immunohistochemistry

All tissue samples had been formalin-fixed and paraffin-embedded. Histological assessment was done using haematoxylin–eosin (HE) stained tissue sections [31]. For the immunostaining, 2 μm thick paraffin sections were cut. The immunoreactions were performed with a non-commercial rabbit polyclonal antibody directed against meprin β (dilution 1:1000). The serum was previously immunised by a peptide produced in *Escherichia coli*, which corresponds to the amino acid sequence of human meprin β from position 450–600. For antigen retrieval, the ER1 antigen retrieval solution was used (30 min). Immunostaining was performed using the Bondmax automatic slide staining system (Leica Biosystems). 3,3′-Diaminobenzidin was used to make immunostaining visible. Counterstaining was performed with hematoxylin.

### Assessment of meprin β staining

**Fig. 1:**
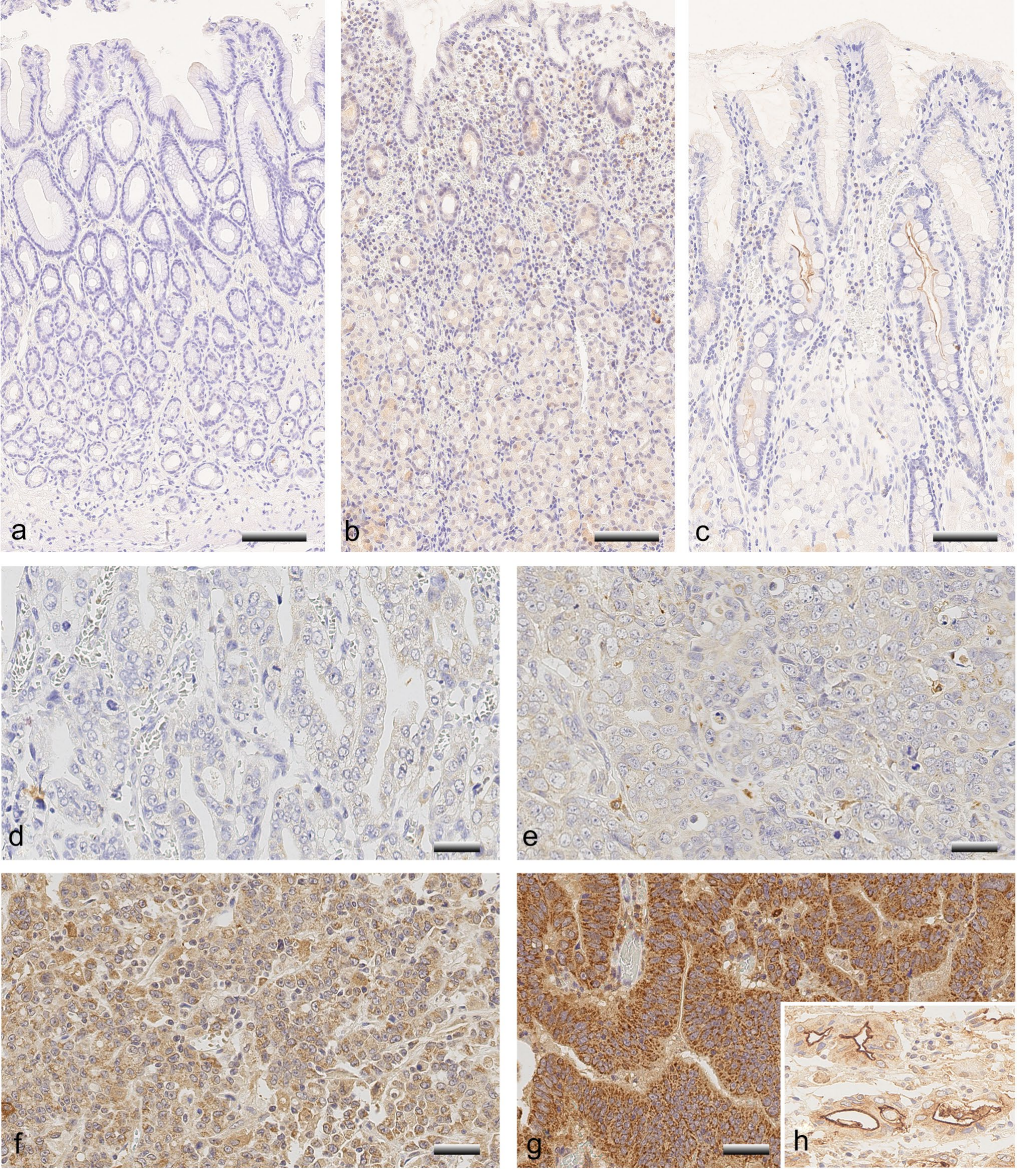
Meprin β immunostaining in non-neoplastic mucosa and gastric carcinoma. Meprin β was not found in foveolar epithelium (**a–c**), antral (**a**) and oxyntic glands (**b**) and was expressed with variable frequency and intensity in intestinal metaplasia (**c**). When assessing staining intensity, these four levels of immunostaining were used as reference sections, i.e., (**d**) represents negative (0), (**e**) weakly positive (1 +), (**f**) moderately positive (2 +), and (**g**) strongly positive (3 +). Membranous staining of tumor cells is shown in (**h**). Anti-meprin β-immunostaining, hemalaun counterstain; 200x- (**a–c**) and 400x (**d–h**) original magnifications; the scale is equivalent to 100 µm (**a**–**c**) and 50 μm (**d–h**)

Immunostaining was evaluated using a Leica Microscope (Leica DM 1000) and the following categories: cytoplasmic and membrane meprin β staining of GC cells, and membrane meprin β staining of intestinal metaplasia and corresponding non-neoplastic mucosa. A 4-level intensity score was used to assess staining intensity, which included the following: negative (0), weakly positive (1 +), moderately (2 +) and strongly (3 +) positive. The stained tissue sections shown in Fig. 1 served as a reference for the different staining intensities (0, 1 + , 2 + , 3 +) during the evaluation of the entire collective.

After screening a whole mount tissue section, the percentage of the respective expression level (0–3) was determined for the categories to be assessed. The sum of the percentages always resulted in 100%, e.g., 10% of the tumor cells showed no immune reaction, 20% of the tumor cells showed the intensity 1 + , 30% 2 + and 40% 3 + . The following formula was then used to calculate the histoscore: Histoscore = [0 × percentage of intensity 0] + [1 × percentage of intensity 1] + [2 × percentage of intensity 2] + [3 × percentage of intensity 3]. The lowest numerical value to be achieved for the histoscore was 0 and the maximum value 300. An example of a 100% strongly coloured preparation would be: [0 × 0%] + [1 × 0%] + [2 × 0%] + [3 × 100%] = 300. By multiplying the higher expression levels by a higher factor, the cases with weak expression were increasingly separated from cases with strong expression. The above example calculates a histoscore of 180 ([0 × 10%] + [1 × 20%] + [2 × 30%] + [3 × 40%] = 180).

### Statistical evaluation

The statistical evaluation was performed with the statistical programme “PASW Statistics” (SPSS) 24.0. The Fisher exact test was used to test the correlation of non-ordinal variables and the Kendall’s tau rank correlation coefficient was used to test the correlation of ordinal variables. We assumed a significance level of 0.05. To compensate for the false discovery rate within the correlations, we applied the Simes (Benjamini-Hochberg) procedure (false discovery rate (FDR)-correction) [32]. Median survival was represented by the Kaplan–Meier survival curves with 95% confidence interval. Differential survival of the “low” and “high” meprin β expressing group was tested by the log rank (Mantel-Cox) test.

## Results

### Study collective

Of the 440 GC patients included, 163 were female (37.0%) and 277 were male (63.0%). The median age at diagnosis was 67.7 years (range 28–92 years). 139 (31.6%) GCs were located in the proximal (esophagogastric junction and cardia) and 298 GCs (67.7%) in the distal stomach. For three patients (0.7%) there was no information about the anatomical location. According to Laurén, 222 (50.0%) patients had an intestinal type, 140 (31.8%) a diffuse type, 31 (7.0%) a mixed type, and 47 GCs (10.7%) were unclassifiable. Patient demographics and the clinicopathological patient characteristics are summarised in Table 1.

**Table 1:** Correlations with clinicopathological patient characteristics

		Total	Meprin β cytoplasmatic score		Meprin β membraneous score		Meprin β intestinal metaplasia score	
			Low	High	Low	High	Low	High
		*n* (%)	*n* (%)	*n* (%)	*n* (%)	*n* (%)	*n* (%)	*n* (%)
Total		n/missing	440/0		440/0		85/355	
		440	219 (49.8)	221 (50.2)	257 (58.4)	183 (41.6)	42 (49.4)	43 (50.6)
Gender	*n* *p*^(1)^	440/0	440	0.767	440	0.368	85	1.000
Male		277 (63.0)	83 (50.9)	80 (49.1)	100 (61.3)	63 (38.7)	14 (48.3)	15 (51.7)
Female		163 (37.0)	136 (49.1)	141 (50.9)	157 (56.7)	120 (43.3)	28 (50.0)	28 (50.0)
Age group	*n* *p*^(1)^	440/0	440	0.446	440	0.333	85	0.824
< 67.7 years		220 (50.0)	114 (51.8)	106 (48.2)	134 (60.9)	86 (39.1)	16 (51.6)	15 (48.4)
≥ 67.7 years		220 (50.0)	105 (47.7)	115 (52.3)	123 (55.9)	97 (44.1)	26 (48.1)	28 (51.9)
Localization	*n* *p*^(1)^	437/3	437	0.258	437	1.000	84	0.713
Proximal		139 (31.8)	75 (54.0)	64 (46.0)	81 (58.3)	58 (41.7)	5 (62.5)	3 (37.5)
Distal		298 (68.2)	142 (47.7)	156 (52.3)	175 (58.7)	123 (41.3)	37 (48.7)	39 (51.3)
Lauren	*n* *p*^(1)^	440/0	440	0.007(*)	440	< 0.001	85	0.747
Intestinal		222 (50.5)	97 (43.7)	125 (56.3)	96 (43.2)	126 (56.8)	26 (53.1)	23 (46.9)
Diffuse		140 (31.8)	76 (54.3)	64 (45.7)	109 (77.9)	31 (22.1)	11 (50.0)	11 (50.0)
Mixed		31 (7.0)	23 (74.2)	8 (25.8)	22 (71.0)	9 (29.0)	2 (33.3)	4 (66.7)
Unclassifiable		47 (10.7)	23 (48.9)	24 (51.1)	30 (63.8)	17 (36.2)	3 (37.5)	5 (62.5)
Grading	*n* *p*^(1)^	222/218	222	0.495	222	0.136	49	0.571
Low grade		96 (43.2)	39 (40.6)	57 (59.4)	36 (37.5)	60 (62.5)	12 (48.0)	13 (52.0)
High grade		126 (56.8)	58 (46.0)	68 (54.0)	60 (47.6)	66 (52.4)	14 (58.3)	10 (41.7)
pT category	*n* *p*^(2)^	440/0	440	0.135	440	0.078	85	0.625
pT1a/T1b		44 (10.0)	21 (47.7)	23 (52.3)	22 (50.0)	22 (50.0)	7 (36.8)	12 (63.2)
pT2		53 (12.0)	21 (39.6)	32 (60.4)	27 (50.9)	26 (49.1)	9 (69.2)	4 (30.8)
pT3		181 (41.1)	90 (49.7)	91 (50.3)	107 (59.1)	74 (40.9)	16 (61.5)	10 (38.5)
pT4a/T4b		162 (36.8)	87 (53.7)	75 (46.3)	101 (62.3)	61 (37.7)	10 (37.0)	17 (63.0)
pN category	*n* *p*^(2)^	439/1	439	0.212	439	0.644	85	0.170
pN0		118 (26.9)	52 (44.1)	66 (55.9)	60 (50.8)	58 (49.2)	19 (54.3)	16 (45.7)
pN1		61 (13.9)	36 (59.0)	25 (41.0)	43 (70.5)	18 (29.5)	5 (62.5)	3 (37.5)
pN2		81 (18.5)	34 (42.0)	47 (58.0)	52 (64.2)	29 (35.8)	9 (56.3)	7 (43.8)
pN3 (a/b)		179 (40.8)	96 (53.6)	83 (46.4)	102 (57.0)	77 (43.0)	9 (34.6)	17 (65.4)
pN plus	*n* *p*^(1)^	440/0	440	0.162	440	0.063	85	0.512
pN0		118 (26.8)	52 (44.1)	66 (55.9)	60 (50.8)	58 (49.2)	19 (54.3)	16 (45.7)
pN1/pN2/pN3 (a/b)		322 (73.2)	167 (51.9)	155 (48.1)	197 (61.2)	125 (38.8)	23 (46.0)	27 (54.0)
pM category	*n* *p*^(1)^	440/0	440	0.472	440	0.144	85	0.351
pM0		354 (80.5)	173 (48.9)	181 (51.1)	213 (60.2)	141 (39.8)	38 (52.1)	35 (47.9)
pM1		86 (19.5)	46 (53.5)	40 (46.5)	44 (51.2)	42 (48.8)	4 (33.3)	8 (66.7)
UICC Stage (8th ed.)	*n* *p*^(2)^	439/1	439	0.382	439	0.672	85	0.505
IA/IB		66 (15.0)	30 (45.5)	36 (54.5)	31 (47.0)	35 (53.0)	10 (41.7)	14 (58.3)
IIA/IIB		97 (22.1)	48 (49.5)	49 (50.5)	59 (60.8)	38 (39.2)	16 (72.7)	6 (27.3)
IIIA/IIIB/IIIC		190 (43.3)	94 (49.5)	96 (50.5)	123 (64.7)	67 (35.3)	12 (44.4)	15 (55.6)
IV		86 (19.6)	46 (53.5)	40 (46.5)	44 (51.2)	42 (48.8)	4 (33.3)	8 (66.7)
Lymph node ratio	*n* *p*^(1)^	439/1	439	0.504	439	0.772	85	0.128
< 0.222		218 (49.7)	100 (47.8)	109 (52.2)	124 (59.3)	85 (40.7)	124 (59.3)	85 (40.7)
> = 0.222		221 (50.3)	118 (51.3)	112 (48.7)	133 (57.8)	97 (42.2)	133 (57.8)	97 (42.2)
pL category	*n* *p*^(1)^	421/19	421	0.770	421	0.373	82	0.374
pL0		199 (47.3)	97 (48.7)	102 (51.3)	112 (56.3)	87 (43.7)	26 (56.5)	20 (43.5)
pL1		222 (52.7)	112 (50.5)	110 (49.5)	135 (60.8)	87 (39.2)	16 (44.4)	20 (55.6)
pV category	*n* *p*^(1)^	420/20	420	0.354	420	0.638	82	0.735
pV0		373 (88.8)	188 (50.4)	185 (49.6)	222 (59.5)	151 (40.5)	38 (52.1)	35 (47.9)
pV1		47 (11.2)	20 (42.6)	27 (57.4)	26 (55.3)	21 (44.7)	4 (44.4)	5 (55.6)
pR status	*n* *p*^(1)^	436/4	436	0.113	436	0.306	84	1.000
pR0		381 (87.4)	183 (48.0)	198 (52.0)	219 (57.5)	162 (42.5)	37 (50.7)	36 (49.3)
pR1		55 (12.6)	33 (60.0)	22 (40.0)	36 (65.5)	19 (34.5)	5 (45.5)	6 (54.5)
H. pylori	*n* *p*^(1)^	377/63	377	0.251	377	1.000	74	1.000
Negative		320 (84.9)	157 (49.1)	163 (50.9)	186 (58.1)	134 (41.9)	34 (53.1)	30 (46.9)
Positive		57 (15.1)	33 (57.9)	24 (42.1)	33 (57.9)	24 (42.1)	5 (50.0)	5 (50.0)
Mucin 1	*n* *p*^(1)^	412/28	412	0.921	412	0.021(*)	79	0.073
Negative		220 (53.4)	108 (49.1)	112 (50.9)	142 (64.5)	78 (35.5)	16 (37.2)	27 (62.8)
Positive		192 (46.6)	96 (50.0)	96 (50.0)	102 (53.1)	90 (46.9)	21 (58.3)	15 (41.7)
Mucin 2	*n* *p*^(1)^	411/29	411	0.184	411	0.468	80	0.817
Negative		262 63.7	136 (51.9)	126 (48.1)	156 (59.5)	106 (40.5)	24 (48.0)	26 (52.0)
Positive		149 (36.3)	67 (45.0)	82 (55.0)	83 (55.7)	66 (44.3)	13 (43.3)	17 (56.7)
Mucin 5	*n* *p*^(1)^	402/38	402	0.425	402	0.686	77	1.000
Negative		208 (51.7)	98 (47.1)	110 (52.9)	122 (58.7)	86 (41.3)	19 (47.5)	21 (52.5)
Positive		194 (48.3)	100 (51.5)	94 (48.5)	109 (56.2)	85 (43.8)	18 (48.6)	19 (51.4)
Mucin 6	*n* *p*^(1)^	416/24	416	0.258	416	0.349	82	0.275
Negative		271 (65.1)	129 (47.6)	142 (52.4)	153 (56.5)	118 (43.5)	25 (54.3)	21 (45.7)
Positive		145 (34.9)	78 (53.8)	67 (46.2)	89 (61.4)	56 (38.6)	15 (41.7)	21 (58.3)
Mucin type	*n* *p*^(1)^	387/53	387	0.106	387	0.033(*)	74	0.884
Intestinal		107 (27.6)	43 (40.2)	64 (59.8)	57 (53.3)	50 (46.7)	8 (42.1)	11 (57.9)
Gastric		60 (15.5)	29 (48.3)	31 (51.7)	38 (63.3)	22 (36.7)	6 (54.5)	5 (45.5)
Mixed		158 (40.8)	85 (53.8)	73 (46.2)	81 (51.3)	77 (48.7)	17 (50.0)	17 (50.0)
Unclassified		62 (16.0)	35 (56.5)	27 (43.5)	44 (71.0)	18 (29.0)	4 (40.0)	6 (60.0)
E-Cadherin	*n* *p*^(1)^	405/35	405	0.821	405	0.011(*)	78	0.237
Negative		299 (73.8)	147 (49.2)	152 (50.8)	187 (62.5)	112 (37.5)	26 (52.0)	24 (48.0)
Positive		106 (26.2)	54 (50.9)	52 (49.1)	51 (48.1)	55 (51.9)	10 (35.7)	18 (64.3)
β-Catenin	*n* *p*^(1)^	406/34	406	0.422	406	< 0.001	76	0.368
Negative		234 (57.6)	119 (50.9)	115 (49.1)	163 (69.7)	71 (30.3)	22 (53.7)	19 (46.3)
Positive		172 (42.4)	80 (46.5)	92 (53.5)	76 (44.2)	96 (55.8)	15 (42.9)	20 (57.1)
HER2 status	*n* *p*^(1)^	414/26	414	0.376	414	0.106	80	0.476
Negative		380 (91.8)	188 (49.5)	192 (50.5)	223 (58.7)	157 (41.3)	38 (52.8)	34 (47.2)
Positive		34 (8.2)	14 (41.2)	20 (58.8)	15 (44.1)	19 (55.9)	3 (37.5)	5 (62.5)
MET status	*n* *p*^(1)^	436/4	436	0.854	436	1.000	85	0.360
Negative		405 (92.9)	200 (49.4)	205 (50.6)	236 (58.3)	169 (41.7)	41 (51.2)	39 (48.8)
Positive		31 (7.1)	16 (51.6)	15 (48.4)	18 (58.1)	13 (41.9)	1 (20.0)	4 (80.0)
Epstein–Barr virus status	*n* *p*^(1)^	426/14	426	1.000	426	0.813	83	0.422
Negative		407 (95.5)	200 (49.1)	207 (50.9)	236 (58.0)	171 (42.0)	36 (46.8)	41 (53.2)
Positive		19 (4.5)	9 (47.4)	10 (52.6)	12 (63.2)	7 (36.8)	4 (66.7)	2 (33.3)
Microsatellite status	*n* *p*^(1)^	425/15	425	0.001	425	0.006(*)	83	0.706
Stable		395 (92.9)	202 (51.1)	193 (48.9)	238 (60.3)	157 (39.7)	36 (47.4)	40 (52.6)
Instable		30 (7.1)	6 (20.0)	24 (80.0)	10 (33.3)	20 (66.7)	4 (57.1)	3 (42.9)
*BRAF* genotype	*n* *p*^(1)^	440/0	440	0.498	440	1.000	85	0.494
Wildtype		439 (99.8)	218 (49.7)	221 (50.3)	256 (58.3)	183 (41.7)	41 (48.8)	43 (51.2)
Mutated		1 (0.2)	1 (100.0)	0 (0.0)	1 (100.0)	0 (0.0)	1 (100.0)	0 (0.0)
*KRAS* genotype	*n* *p*^(1)^	440/0	440	0.446	440	0.001	85	1.000
Wildtype		424 (96.4)	213 (50.2)	211 (49.8)	254 (59.9)	170 (40.1)	41 (49.4)	42 (50.6)
Mutated		16 (3.6)	6 (37.5)	10 (62.5)	3 (18.8)	13 (81.3)	1 (50.0)	1 (50.0)
*PIK3CA* genotype	*n* *p*^(1)^	440/0	440	0.656	440	1.000	85	0.360
Wildtype		419 (95.2)	210 (50.1)	209 (49.9)	245 (58.5)	174 (41.5)	39 (48.1)	42 (51.9)
Mutated		21 (4.8)	9 (42.9)	12 (57.1)	12 (57.1)	9 (42.9)	3 (75.0)	1 (25.0)
PD-L1 in tumor cells	*n* *p*^(1)^	420/20	420	0.038(*)	420	0.035(*)	79	0.323
Negative (IRS ≤ 2)		321 (76.4)	170 (53.0)	151 (47.0)	199 (62.0)	122 (38.0)	26 (45.6)	31 (54.4)
Positive (IRS > 2)		99 (23.6)	40 (40.4)	59 (59.6)	49 (49.5)	50 (50.5)	13 (59.1)	9 (40.9)
PD-L1 in immune cells	*n* *p*^(1)^	420/20	420	0.543	420	0.758	79	0.232
Negative (Quantity Score ≤ 1)		267 (63.6)	137 (51.3)	130 (48.7)	156 (58.4)	111 (41.6)	24 (44.4)	30 (55.6)
Positive (Quantity Score > 1)		153 (36.4)	73 (47.7)	80 (52.3)	92 (60.1)	61 (39.9)	15 (60.0)	10 (40.0)
PD-1 in immune cells	*n* *p*^(1)^	423/17	423	0.284	423	0.921	79	0.263
Not present		194 (45.9)	91 (46.9)	103 (53.1)	115 (59.3)	79 (40.7)	22 (56.4)	17 (43.6)
Present		229 (54.1)	120 (52.4)	109 (47.6)	134 (58.5)	95 (41.5)	17 (42.5)	23 (57.5)
VISTA	*n* *p*^(1)^	421/19	421	0.610	421	0.383	79	1.000
Negative		384 (91.2)	194 (50.5)	190 (49.5)	229 (59.6)	155	(40.4)	38 (50.0)
Positive		37 (8.8)	17 (45.9)	20 (54.1)	19 (51.4)	18 (48.6)	1 (33.3)	2 (66.7)
Overall survival [months]	*p*^(3)^			0.011(*)		0.661		0.147
Total/events/censored		428/338/90	213/179/34	215/159/56	250/195/55	178/143/35	40/28/12	40/30/10
Median Survival		14.1 ± 1.0	14.1 ± 1.5	14.0 ± 1.6	16.0 ± 1.9	12.7 ± 1.2	20.1 ± 10.2	11.9 ± 2.4
95% CI		12.1–16.0	12.0–17.0	11.0–17.0	12.2–19.8	10.5–15.0	0.1–40.2	7.1–16.6
Tumor-specific survival [months]	*p*^(3)^			0.005(*)		0.806		0.256
Total/events/censored		402/280/122	197/150/47	205/130/75	236/166/70	166/114/52	37/19/18	38/21/17
Median survival		15.5 ± 1.3	14.6 ± 1.7	16.6 ± 3.1	16.8 ± 2.2	14.0 ± 1.3	38.0 ± 17.1	14.7 ± 3.6
95% CI		13.0–18.0	11.2–18.0	10.5–22.7	12.5–21.1	11.4–16.7	4.5–71.5	7.5–21.8

^(1)^Fisher’s exact test

^(2)^Kendall’s tau

^(3)^Log-rank test

^(^*^)^Not significant after multiple testing correction

### Meprin β is expressed in gastric cancer and intestinal metaplasia

Immunohistochemistry showed that meprin β is expressed by tumor cells at the cell membrane and/or in the cytoplasm (Fig. 1), and also in non-neoplastic mucosa, here particularly in the intestinal metaplasia. Nuclear expression was not found.

Cytoplasmic expression in GC was found in 440 cases. Staining intensity varied from 0 to 3 + . The medium histoscore was 92 (range 0–170). 328 of these cases had a combination of different staining intensities.

Membranous expression of meprin β in GC was found in 163 cases. The medium histoscore was 0 (range 0–145). 83 of these cases had a combination of different staining intensities.

Corresponding non-neoplastic mucosa was assessable in 116 cases. Among these, 85 showed an intestinal metaplasia and 33 unaltered gastric mucosa, i.e., without intestinal metaplasia and without dysplasia. Gastric foveolar epithelium was always immunonegative for meprin β, as were the glands of antral and oxyntic mucosa (Fig. 1). Membranous expression of meprin β in the intestinal metaplasia of the non-neoplastic mucosa was observed in 123 cases. The medium histoscore was 150 (range 0–250). 79 of these cases had a combination of different staining intensities (Fig. 1).

Collectively these data show that meprin β is differentially and heterogeneously expressed in GC tissue and adjacent non-neoplastic stomach mucosa.

### Correlations with clinicopathological patient characteristics

To test for a correlation between meprin β expression and numerous clinicopathological patient characteristics, the histoscore for tumor cell cytoplasmic, tumor cell membranous and intestinal metaplasia characteristics was dichotomised into a “meprin β negative/low” versus “meprin β positive/high” group at the respective median (see above).

Table 1 summarises the correlations of meprin β expression in tumor cells and intestinal metaplasia with various clinicopathological patient characteristics.

#### Correlation of cytoplasmic expression of meprin β with clinicopathological patient characteristics

Cytoplasmic expression of meprin β positive/high was found more frequently in intestinal type and significantly more frequently in microsatellite instable GCs (*p* = 0.001; Table 1). PD-L1 expression on tumor cells was divided into two groups based on the immunoreactivity score (IRS). All tumors with an IRS less than or equal to 2 (321 GCs) were negative and those with an IRS greater than 2 (99 GCs) were positive. High cytoplasmic stainability for meprin β was found to be associated with positive PD-L1 IRS (59.6% vs. 47.0%; Table 1).

None of the other clinicopathological parameters correlated with cytoplasmic expression.

#### Correlation of membranous expression of meprin β with clinicopathological patient characteristics

Membranous expression of meprin β positive/high was found significantly more frequently in intestinal type GCs (*p* < 0.001; Table 1). Furthermore, meprin β-positive/high expression correlated with the mucin phenotype (more common in the intestinal and with mixed type) and was more frequent in mucin-1-positive GCs. Interestingly, positive/high membrane meprin β expression was associated with positive E-cadherin- (*p* = 0.011; not significant after multiple testing correction) and β-catenin status (*p* < 0.001) (Table 1) and more commonly found in microsatellite instable GCs, and *KRAS* mutated GCs (*p* = 0.001), respectively. Again, PD-L1 expression was associated with meprin β expression, i.e., PD-L1-positive GCs were more frequently meprin β positive (50.5% vs. 38.0%; Table 1).

None of the other clinicopathological parameters correlated with membranous expression.

The expression of meprin β in the intestinal metaplasia did not correlate with any clinicopathological patient characteristic (Table 1).

### Survival analysis

**Fig. 2:**
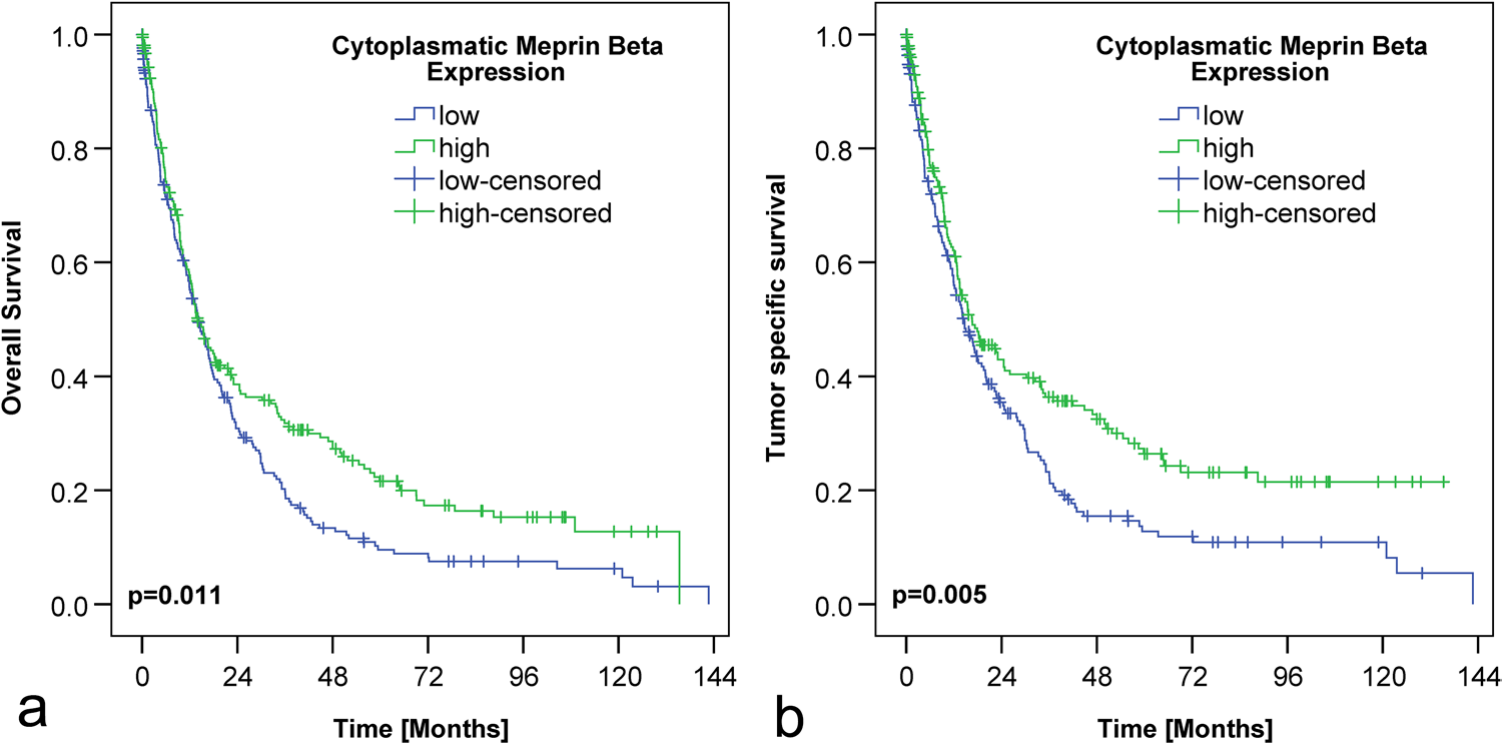
Meprin β and survival in cytoplasmic expression in gastric carcinoma. Shown as Kaplan–Meier survival curves. X-axis: survival in months, Y-axis: cumulative survival. Significant correlations were shown in overall survival (**a**) and tumor-specific survival (**b**). Blue graph: meprin β “low” (Histoscore < 92), Green graph: meprin β “high” (histoscore ≥ 92)

Finally, we compared the dichotomized expression of cytoplasmic and membranous expression of the meprin β with overall and tumor-specific survival. Patients with positive/high cytoplasmic expression of meprin β had a better overall and tumor-specific survival (Table 1; Fig. 2). Membranous expression of meprin β did not correlate with overall or tumor-specific survival (Table 1). The multivariate survival analysis showed that meprin β is not an independent predictor of survival.

## Discussion

Gastric cancer is the prototype of inflammation-associated carcinogenesis, where colonisation of the stomach mucosa with HP is the strongest known risk factor [3]. In general, each area of the body has a preferred microbiome, and altering its composition can lead to dysbiosis and inflammation [3]. Apart from HP, increasing evidence shows that the microbiome, in more general, plays a key role in tumor progression of GC by regulating the immune response in a complex manner and by inflammatory events supporting carcinogenesis [3]. The interactions between microbes, the physiological microbiome and cellular processes and signalling pathways are diverse [3].

Here, we explored the expression and putative tumor biological role of meprin β in GC. To the best of our knowledge, we are the first to illustrate the differential expression of meprin β in GC. It was found in the cytoplasm and at the cell membrane and correlated with diverse clinicopathological patient characteristics, including tumor type according to the Lauren, mucin phenotype, microsatellite status, E-cadherin and β-catenin expression, *KRAS* genotype and PD-L1 status in tumor cells. Furthermore, cytoplasmic expression of meprin β was associated with better patient outcome. Meprin ß was absent in non-neoplastic gastric foveolar epithelium and found in intestinal metaplasia. Collectively, these data show that meprin β is differentially expressed in GC and of putative tumor biological significance.

### Meprin β may function as a tumor suppressor in gastric carcinoma

The expression of meprin β was lower in locally advanced GCs and consequently has less anti-inflammatory and anti-carcinogenic effect, probably through mucus cleavage, assuming analogous functioning as in the small intestine. These findings support the contention that meprin β functions as a tumor suppressor in GC and is lost during local tumor progression. The “protective” effect of meprin β might also explain its higher expression in intestinal type GC, which is associated with a better prognosis compared to the other Lauren phenotypes [23], and the differential expression of mucins, which also correlate with patient prognosis [23].

Mucin 1 is membrane-bound on the apical side of the cell membrane and also diffusely in the cytoplasm [33]. It has a protective function in the normal gastric mucosa, protecting the gastric epithelium from a variety of external influences that are causative for inflammation and carcinogenesis [33]. On the contrary, mucin 1 is considered an oncogene with anti-apoptotic function in tumor cells. In addition, the gene locus of mucin 1 is a locus for increased susceptibility to the development of GC [33, 34]. Expression of mucin 1 in tumor cells is associated with poor prognosis [34].

In our collective, the occurrence of mucin 1 correlated with a high membrane-bound meprin β expression, which in turn occurs mainly in early tumor stages. These data could be interpreted in a way that mucin 1 might maintain its tumor-suppressive function in early tumor stages and turns it into the opposite during tumor progression. Membranous expression of meprin β also correlated with the mucin phenotype, which supports the contention that the effect of meprin β and mucins on GC biology seem to be interconnected.

### The correlation of meprin β with E-cadherin and β-catenin points towards context-dependent pathophysiological mechanisms in gastric cancer biology

E-cadherin is a transmembrane glycoprotein with an essential role in calcium-dependent cell–cell adhesion. It, therefore, has an important influence on epithelial architecture and maintains cell polarity and differentiation [35, 36]. It is also one of the tumor suppressors. In GC, its function is downregulated, e.g., by mutations in the gene locus of E-cadherin and epigenetic factors, such as DNA hypermethylation. Dysregulation of the glycoprotein occurs mainly in the Lauren diffuse type and leads to gastric epithelial cell dysfunction and tumor progression and invasion [35, 36].β-Catenin, in turn, is an intracellular scaffold protein that interacts with adhesion molecules, such as E-cadherin, with transmembrane mucins, such as mucin 1, with signalling regulators and with epigenetic or transcriptional regulators [37, 38]. In general, dysregulation of β-catenin signalling pathways is associated with chronic inflammation, fibrosis and various cancers [37]. In addition, the activation of β-catenin is associated with HP infection and abnormal β-catenin expression correlates with tumor progression in GC [39].

High/positive expression of meprin β correlated with both, a positive E-cadherin- and a positive β-catenin status. The correlation with the tumor suppressor E-cadherin and its correlation with the histological phenotype, like it was also found for E-cadherin, fits into the putative tumor-suppressive role of meprin β. Thus, the expression patterns of E-cadherin and meprin β are concordant. The correlation of meprin β with β-catenin points towards an opposite pathophysiological mechanism of meprin β. Thus, meprin β may have divergent, context-dependent effects in GC biology.

### The correlation with PD-L1 indicates that meprin β is also involved in cancer immunology

PD-1 is an immune checkpoint protein that binds PD-L1 and PD-L2 as ligands, triggers programmed cell death of T- and B cells through this binding and in this way regulates their proliferation [20, 40]. In malignant tumors, the ligands PD-L1 and PD-L2 are upregulated, bind to the PD-1 receptor on the cell surface of T cells and consequently inhibit T cell activity. The T cell response against tumor cells is dampened and allows the tumor to escape the immune response [20, 40]. PD-L1 is more highly expressed in GC than in the corresponding normal tissue [41].

The correlation of high membrane and high cytoplasmic meprin β expression with positive IRS in PD-L1 expression on tumor cells may indicate that meprin β plays also a role in immune evasion of GC. In support of this contention, a high membrane and high cytoplasmic meprin β expression was also more commonly found in MSI GCs, which frequently upregulate PD-L1 in order to evade immune destruction due to high neoantigen load [20].

## Conclusion

Our study shows that meprin β is differentially expressed in GC and correlates with Lauren phenotype, mucins status, E-cadherin-, β-catenin-, MSI- and PD-L1 status. While reduced expression is associated with worse patient outcome, meprin β may have pleiotropic effects on GC biology. An underlying mechanism behind the tumor-suppressive effect of meprin β could be, e.g., the prevention of bacterial overgrowth by mucin cleavage and the resulting anti-inflammatory effect [10]. Meprin β may counteract tumor progression through its anti-inflammatory effect and reduced expression might decrease mucus cleavage. In the absence of mucus shedding, the mucus might not be longer loosely attached and rapidly renewed as would be necessary for efficient intestinal barrier function and prevention of bacterial overgrowth, but the mucus is tightly packed and firmly attached to the epithelium. As a result, intestinal barrier function is impaired, promoting further bacterial colonisation and dysbiosis of the mucosa. The alteration of the microbiome could additionally influence meprin β, as for example the pathogen, *P. gingivalis*, secretes the protease Arg-gingipain (RgpB), which converts the membrane-bound meprin β into its active form [19]. This activation prevents meprin β cleavage and thus impairs the anti-inflammatory function of meprin β as a mucus-releasing protease [19]. In addition, activated membrane-bound meprin β cleaves the IL-6 receptor and CD99 [10]. The release of the IL-6 receptor activates IL-6 trans-signalling and the release of CD99 provides increased transendothelial cell migration. Both mechanisms have a proinflammatory effect [10]. In addition, IL-6 trans-signalling promotes proliferation, invasion and metastasis of GC cells [13]. Furthermore, it induces increased VEGF-C production via the JAK-STAT3 signalling pathway, which results in increased lymphangiogenesis and consequently improves blood supply to the carcinoma [13]. For IL-6, a positive correlation was found with lymph node metastasis and correspondingly a negative correlation with survival of patients with GC [13]. It would be conceivable that *P. gingivalis* and possibly other pathogens activate meprin β membrane-bound and consequently reduce physiological anti-inflammatory function through mucus cleavage and promote proinflammatory and pro-carcinogenic effects through cleavage of the IL-6 receptor and CD99. Collectively, these data support the contention that meprin β is involved in GC biology. Meprin β, the microbiome and gastric mucosal inflammation might influence each other [3]. A tumor biological relevance of meprin β in GC is highly probable and further research on this topic is warranted.

## Data Availability

The data presented in this study are available on request from the corresponding author.

## References

[1] Camargo MC, Figueiredo C, Machado JC (2019). Review: gastric malignancies: basic aspects. Helicobacter.

[2] Robert-Koch-Institut. Zentrum für Krebsregisterdaten—Magenkarzinom. https://www.krebsdaten.de/Krebs/EN/Content/Cancer_sites/Stomach_cancer/stomach_cancer_node.html. 2019.

[3] Robertson, E.S., Microbiome and Cancer. Current Cancer Research, ed. W. El-Deiry. 2019, Gewerbestrasse 11, 6330 Cham, Switzerland. 401

[4] Smyth, et al. Gastric cancer. Lancet. 2020;396(10251):635–48. 10.1016/S0140-6736(20)31288-5. 10.1016/S0140-6736(20)31288-5 32861308

[5] Ferreira RM (2018). Gastric microbial community profiling reveals a dysbiotic cancer-associated microbiota. Gut.

[6] Wang F (2014). Helicobacter pylori-induced gastric inflammation and gastric cancer. Cancer Lett.

[7] Wroblewski LE, Peek RM (2016). Helicobacter pylori, cancer, and the gastric microbiota. Adv Exp Med Biol.

[8] Helmink BA (2019). The microbiome, cancer, and cancer therapy. Nat Med.

[9] Ebrahimi V (2020). Epigenetic modifications in gastric cancer: focus on DNA methylation. Gene.

[10] Arnold P, Otte A, Becker-Pauly C (2017). Meprin metalloproteases: Molecular regulation and function in inflammation and fibrosis. Biochim Biophys Acta.

[11] Jefferson T (2013). The substrate degradome of meprin metalloproteases reveals an unexpected proteolytic link between meprin beta and ADAM10. Cell Mol Life Sci.

[12] Arnold P (2017). Meprin metalloproteases generate biologically active soluble interleukin-6 receptor to induce trans-signaling. Sci Rep.

[13] Zhao G (2016). IL-6 mediates the signal pathway of JAK-STAT3-VEGF-C promoting growth, invasion and lymphangiogenesis in gastric cancer. Oncol Rep.

[14] Prox J, Arnold P, Becker-Pauly C (2015). Meprin alpha and meprin beta: Procollagen proteinases in health and disease. Matrix Biol.

[15] Werny L (2023). MT1-MMP and ADAM10/17 exhibit a remarkable overlap of shedding properties. FEBS J.

[16] Broder C (2013). Metalloproteases meprin alpha and meprin beta are C- and N-procollagen proteinases important for collagen assembly and tensile strength. Proc Natl Acad Sci U S A.

[17] Schutte A (2014). Microbial-induced meprin beta cleavage in MUC2 mucin and a functional CFTR channel are required to release anchored small intestinal mucus. Proc Natl Acad Sci USA.

[18] Jackle F (2015). Metalloprotease meprin beta is activated by transmembrane serine protease matriptase-2 at the cell surface thereby enhancing APP shedding. Biochem J.

[19] Wichert R (2017). Mucus detachment by host metalloprotease meprin beta requires shedding of its inactive pro-form, which is abrogated by the pathogenic protease RgpB. Cell Rep.

[20] Böger C (2016). PD-L1 is an independent prognostic predictor in gastric cancer of Western patients. Oncotarget.

[21] Lauren P (1965). The two histological main types of gastric carcinoma: diffuse and so-called intestinal-type carcinoma. An attempt at a histo-clinical classification. Acta Pathol Microbiol Scand.

[22] O'Sullivan B (2017). The TNM classification of malignant tumours-towards common understanding and reasonable expectations. Lancet Oncol.

[23] Warneke VS (2013). Prognostic and putative predictive biomarkers of gastric cancer for personalized medicine. Diagn Mol Pathol.

[24] Böger C (2017). Epstein-Barr virus-associated gastric cancer reveals intratumoral heterogeneity of PIK3CA mutations. Ann Oncol.

[25] Mathiak M (2017). Clinicopathologic characteristics of microsatellite instable gastric carcinomas revisited: urgent need for standardization. Appl Immunohistochem Mol Morphol.

[26] Warneke VS (2013). Members of the EpCAM signalling pathway are expressed in gastric cancer tissue and are correlated with patient prognosis. Br J Cancer.

[27] Warneke VS (2013). Her2/neu testing in gastric cancer: evaluating the risk of sampling errors. Ann Oncol.

[28] Metzger ML (2016). MET in gastric cancer–discarding a 10% cutoff rule. Histopathology.

[29] Böger C (2017). The novel negative checkpoint regulator VISTA is expressed in gastric carcinoma and associated with PD-L1/PD-1: a future perspective for a combined gastric cancer therapy?. Oncoimmunology.

[30] Schoop H (2020). Therapy resistance in neoadjuvantly treated gastric cancer and cancer of the gastroesophageal junction is associated with an increased expression of immune checkpoint inhibitors-comparison against a therapy naive cohort. Transl Oncol.

[31] Lüllmann-Rauch R (2012). Taschenlehrbuch histologie.

[32] Simes RJ (1986). An improved Bonferroni procedure for multiple tests of significance. Biometrika.

[33] Saeki N, Sakamoto H, Yoshida T (2014). Mucin 1 gene (MUC1) and gastric-cancer susceptibility. Int J Mol Sci.

[34] Wang XT (2016). MUC1 immunohistochemical expression as a prognostic factor in gastric cancer: meta-analysis. Dis Mark.

[35] Bure IV, Nemtsova MV, Zaletaev DV (2019). Roles of E-cadherin and noncoding RNAs in the epithelial-mesenchymal transition and progression in gastric cancer. Int J Mol Sci.

[36] Liu X, Chu KM (2014). E-cadherin and gastric cancer: cause, consequence, and applications. Biomed Res Int.

[37] Katoh M (2018). Multilayered prevention and treatment of chronic inflammation, organ fibrosis and cancer associated with canonical WNT/betacatenin signaling activation (Review). Int J Mol Med.

[38] Valenta T, Hausmann G, Basler K (2012). The many faces and functions of beta-catenin. EMBO J.

[39] Li L, et al. Abnormal β-catenin immunohistochemical expression as a prognostic factor in gastric cancer: a meta-analysis. World J Gastroenterol. 2014;20(34):12313–21. 10.3748/wjg.v20.i34.12313 PMC4161818 25232267

[40] Cui C (2019). The roles of PD-1/PD-L1 and its signalling pathway in gastrointestinal tract cancers. Clin Exp Pharmacol Physiol.

[41] Sun L (2018). Gastric cancer mesenchymal stem cells derived IL-8 induces PD-L1 expression in gastric cancer cells via STAT3/mTOR-c-Myc signal axis. Cell Death Dis.

